# Mouse Chromosome 4 Is Associated with the Baseline and Allergic IgE Phenotypes

**DOI:** 10.1534/g3.117.042739

**Published:** 2017-07-10

**Authors:** Cynthia Kanagaratham, Pierre Camateros, John Ren, Robert Sladek, Silvia M. Vidal, Danuta Radzioch

**Affiliations:** * Department of Human Genetics, McGill University, Montreal, Quebec, H3A 1B1, Canada; † Division of Experimental Medicine, Department of Medicine, Faculty of Medicine, McGill University, Montreal, Quebec, H4A 3J1, Canada; § Department of Microbiology and Immunology; ** Institute of Parasitology, McGill University, Montreal, Quebec, H9X 3V9, Canada; †† Genome Quebec Innovation Centre, McGill University, Montreal, Quebec, H3A 0G1, Canada

**Keywords:** animal models, allergy, QTL mapping, complex traits, IgE

## Abstract

Regulation of IgE concentration in the blood is a complex trait, with high concentrations associated with parasitic infections as well as allergic diseases. A/J strain mice have significantly higher plasma concentrations of IgE, both at baseline and after ovalbumin antigen exposure, when compared to C57BL/6J strain mice. Our objective was to determine the genomic regions associated with this difference in phenotype. To achieve this, we used a panel of recombinant congenic strains (RCS) derived from A/J and C57BL/6J strains. We measured IgE in the RCS panel at baseline and following allergen exposure. Using marker by marker analysis of the RCS genotype and phenotype data, we identified multiple regions associated with the IgE phenotype. A single region was identified to be associated with baseline IgE level, while multiple regions wereassociated with the phenotype after allergen exposure. The most significant region was found on Chromosome 4, from 81.46 to 86.17 Mbp. Chromosome 4 substitution strain mice had significantly higher concentration of IgE than their background parental strain mice, C57BL/6J. Our data presents multiple candidate regions associated with plasma IgE concentration at baseline and following allergen exposure, with the most significant one located on Chromosome 4.

Plasma IgE antibodies are commonly associated with allergic disorders, and are often elevated in patients with allergic asthma (Jackola *et al.* 2004). Epidemiological studies have shown that elevated plasma IgE level is a risk factor for asthma (Ishizaka and Ishizaka 1971), and results from family and twin studies indicate that regulation of circulating IgE levels is largely genetically determined (Bazaral *et al.* 1971). Best fitting model studies have attributed several possible modes of inheritance for the phenotype including polygenic and recessive, dominant and codominant (Gerrard *et al.* 1978; Meyers *et al.* 1982, 1991; Hasstedt *et al.* 1983; Martinez *et al.* 1994; Dizier *et al.* 1995). Linkage and genome wide association studies in humans have found associations between serum concentrations of IgE and various loci, with the most replicated loci being *STAT6* (on Chromosome 12) and *IL-13* (on Chromosome 5) (Vercelli 2008; Potaczek and Kabesch 2012).

Few genetic studies for IgE levels have been done using animal models, and they were focused on the IgE concentration after exposure to parasites (Badalova *et al.* 2002; Lipoldova *et al.* 2002; Menge *et al.* 2003). Examples include association studies for the IgE phenotype in response to *Leishmania major* infection in a BALB/c × STS/A recombinant congenic panel (Lipoldova *et al.* 2000; Badalova *et al.* 2002), in response to *Heligmosomoides polygyrus* resistance in a CBA × SWR strain cross (Menge *et al.* 2003), and in response to ovalbumin allergen sensitization and challenge in an SM/J × A/J recombinant inbred strain panel (Ohno *et al.* 2013). These studies all investigated the IgE phenotype following antigen exposure. We believe that studying the genetic signature of baseline levels of IgE (prior to allergen exposure), and how the signature is changed following allergen exposure, can provide valuable information about the innate predisposition of expressing heightened levels of IgE, and the modulation of IgE levels following allergen exposure. This would help determine if an allergy susceptibility region can be identified even before allergen exposure. In humans, baseline levels of IgE have been shown to be a predictor of long-term disease outcome, such as in patients with atopic dermatitis (Kiiski *et al.* 2015). Furthermore, it has been shown in certain populations that sensitization to environmental allergens, increasing baseline levels of plasma IgE, is a risk factor for the development of asthma, other allergic disorders, and lung infections (Leung *et al.* 1997; Skaaby *et al.* 2017).

The present study is aimed at identifying regions associated with IgE concentration at baseline, following allergen sensitization, and following allergen sensitization and challenge. To achieve this, we used an AcB/BcA panel of recombinant congenic strains (RCS), and an ovalbumin induced model of allergic asthma. The AcB/BcA panel of RCS was derived from the parental strains A/J and C57BL/6J, two strains with significantly differing IgE phenotypes. To date, these strains have been used in studies on topics such as resistance to infectious diseases like influenza and *Salmonella*, psychiatric disorders and addiction, and lung responsiveness (Gill and Boyle 2005; Roy *et al.* 2006; Camateros *et al.* 2010; Boivin *et al.* 2012). Each RCS is fully inbred, and composed of ∼12.5% of genetic material from one parental strain (minor genetic donor) and ∼87.5% from the other (major genetic donor) (Fortin *et al.* 2001). Here, we use this panel to identify the genomic regions associated with levels of circulating IgE at baseline, and after antigen exposure.

## Materials and Methods

### Mice

Breeding pairs for A/J, C57BL/6J, C57BL/6J-Chr 4A/J/NaJ (CSS4), and C57BL/6J-Chr 12A/J/NaJ (CSS12) mice were purchased from Jackson Laboratories (Boston, MA). The AcB/BcA panel of RCS were generated at the Montreal General Hospital Research Institute from A/J and C57BL/6J mice (Fortin *et al.* 2001). All mice were bred and housed at the Montreal General Hospital Research Institute animal facility in specific pathogen-free conditions with a 12-hr light/dark cycle. Animals were housed at a maximum of five animals per cage, and had *ad libitum* access to food and water. Only male mice aged 9–12 wk were used in all experiments. Animal protocols were approved by the Montreal General Hospital Facility Animal Care Committee, and were in compliance with the regulations set by the Canadian Council for Animal Care.

### Allergic model and sample collection

To generate the allergic model, mice were sensitized by three weekly intraperitoneal injections of 100 µg of ovalbumin (Sigma) adsorbed to 1.5 mg of aluminum hydroxide (Imject Alum, Pierce), in a total volume of 0.2 ml phosphate buffered saline (PBS). At 1 wk following the third sensitization, animals were aerosol challenged for 3 d consecutively with a 30-min exposure to either 1% ovalbumin solution or PBS. At 3 hr after the final challenge, mice were killed by CO_2_ exposure, and blood was collected by cardiac puncture in EDTA-coated tubes. Blood samples were spun at 3000 rpm for 7 min at 4° to isolate the plasma.

### IgE measurements

Total IgE in the plasma was measured by enzyme-linked immunosorbent assay (ELISA) using the mouse BD OptEIA kit (BD Biosciences) following the manufacturer’s instructions.

### Identification of significantly associated regions

A marker-by-marker analysis was conducted to identify associations between genotype and phenotype, as previously described (Camateros *et al.* 2010). Each phenotype was analyzed individually using log2-transformed phenotype data, and an established list of 1215 single nucleotide polymorphism (SNP) and microsatellite markers (Boivin *et al.* 2012). The analysis was done using the statistical software R, version 3.3.2. Manhattan plots were produced using the qqman package for R (Turner 2014).

### Statistical analysis

Unless otherwise specified, data were analyzed by GraphPad version 5.03 (GraphPad Software Inc.). One-way ANOVA followed by a Dunnet’s post-test procedure was used to compare each RCS to its respective major parental strains. All other comparisons between three groups was done by one-way ANOVA followed by Bonferroni post-test. *P* values ≤0.05 were considered to be significant.

### Data availability

Supplemental Material, File S1, contains the phenotyping data of the RCS and parental strains. Genotyping marker ID and location, along with genotype for each strain, has been previously published (Boivin *et al.* 2012, File S1).

## Results

### IgE phenotype in RCS panel with increased antigen exposure

**Figure 1:**
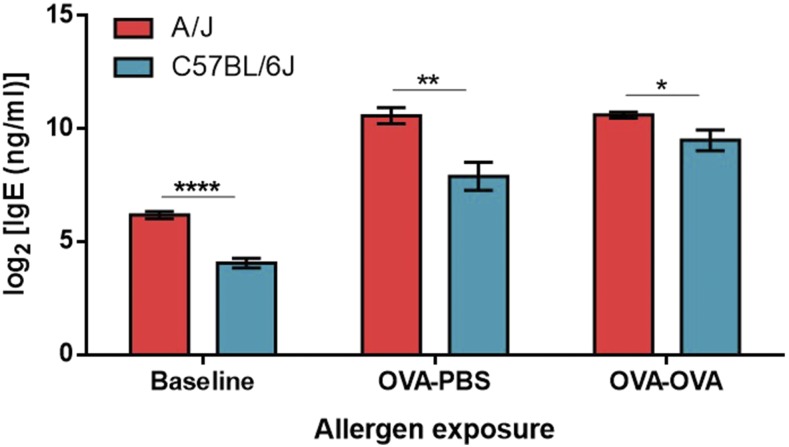
Comparison of plasma IgE levels in A/J and C57BL/6J mice at baseline and following allergen exposure. The A/J strain has a higher IgE concentration in all three conditions compared to the C57BL/6J strain. Results are presented for unsensitized (Baseline), ovalbumin (OVA) sensitized and PBS challenged (OVA-PBS), and OVA sensitized and OVA challenged animals (OVA-OVA). Data are presented as mean of log_2_ transformed values ± SEM. Significant differences between strains was determined by a two-tailed *t*-test. *n* > 6 per strain. *, **, and *** represent *P* < 0.05, *P* < 0.01, and *P* < 0.001, respectively.

**Figure 2:**
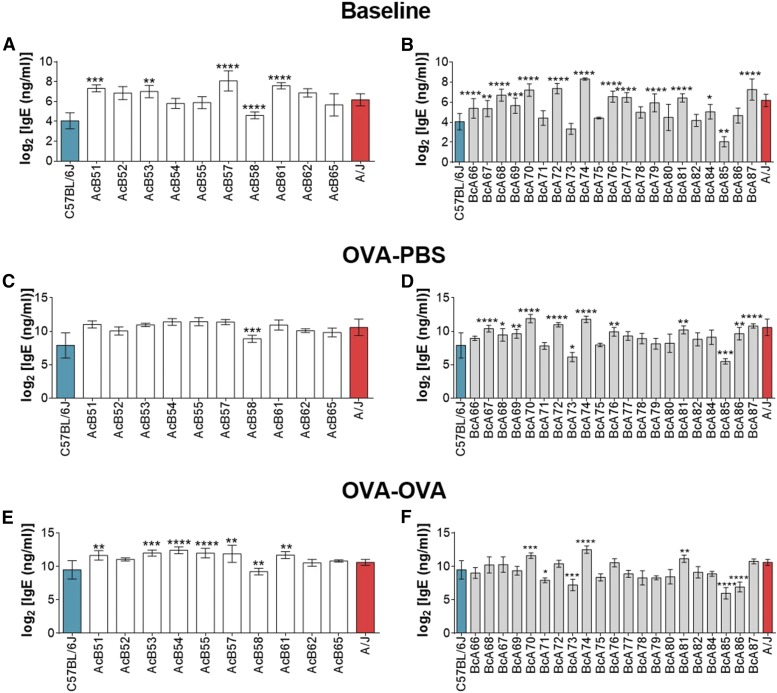
Distribution of the IgE phenotype in the RCS compared to parental strains. AcB strains were compared to A/J strain, while BcA strains were compared to C57BL/6J strain. (A, B) Baseline; (C, D) (OVA-PBS) ovalbumin (OVA) sensitized; (E, F) OVA sensitized and OVA challenged animals (OVA-OVA). Data are presented as mean of log_2_ transformed values ± SEM. Comparisons between RCS and respective major genetic donor parental strains were done by one-way ANOVA followed by Dunnett’s correction. *n* = 5–20 per strain, 724 mice in total. *, **, and *** represent *P* < 0.05, *P* < 0.01, and *P* < 0.001, respectively.

Previous studies have demonstrated that A/J and C57BL/6J strains of mice have significantly different levels of plasma IgE concentrations (Figure 1) (Moisan *et al.* 2006). Our goal was to uncover the genetic determinants that may cause this difference between these two strains, both at baseline and after antigen exposure. To achieve this, plasma IgE concentration was measured from each of the 10 AcB and 21 BcA strains of the RCS panel derived from the A/J and C57BL/6J parental strains. IgE was measured in all strains at baseline (Figure 2, A and B), after sensitization to ovalbumin (Figure 2, C and D), and after sensitization and aerosol challenged with ovalbumin (Figure 2, E and F). Informative strains, whose phenotypes are significantly different from their major parental strain, are mostly from the BcA family, and they most likely contain the segregating alleles influencing the IgE trait. Important informative strains include BcA85, which has the lowest IgE phenotype in all three models, and BcA74, which has one of the highest phenotypes. The AcB strain, with the lowest phenotype, is AcB58.

### Marker association

**Figure 3:**
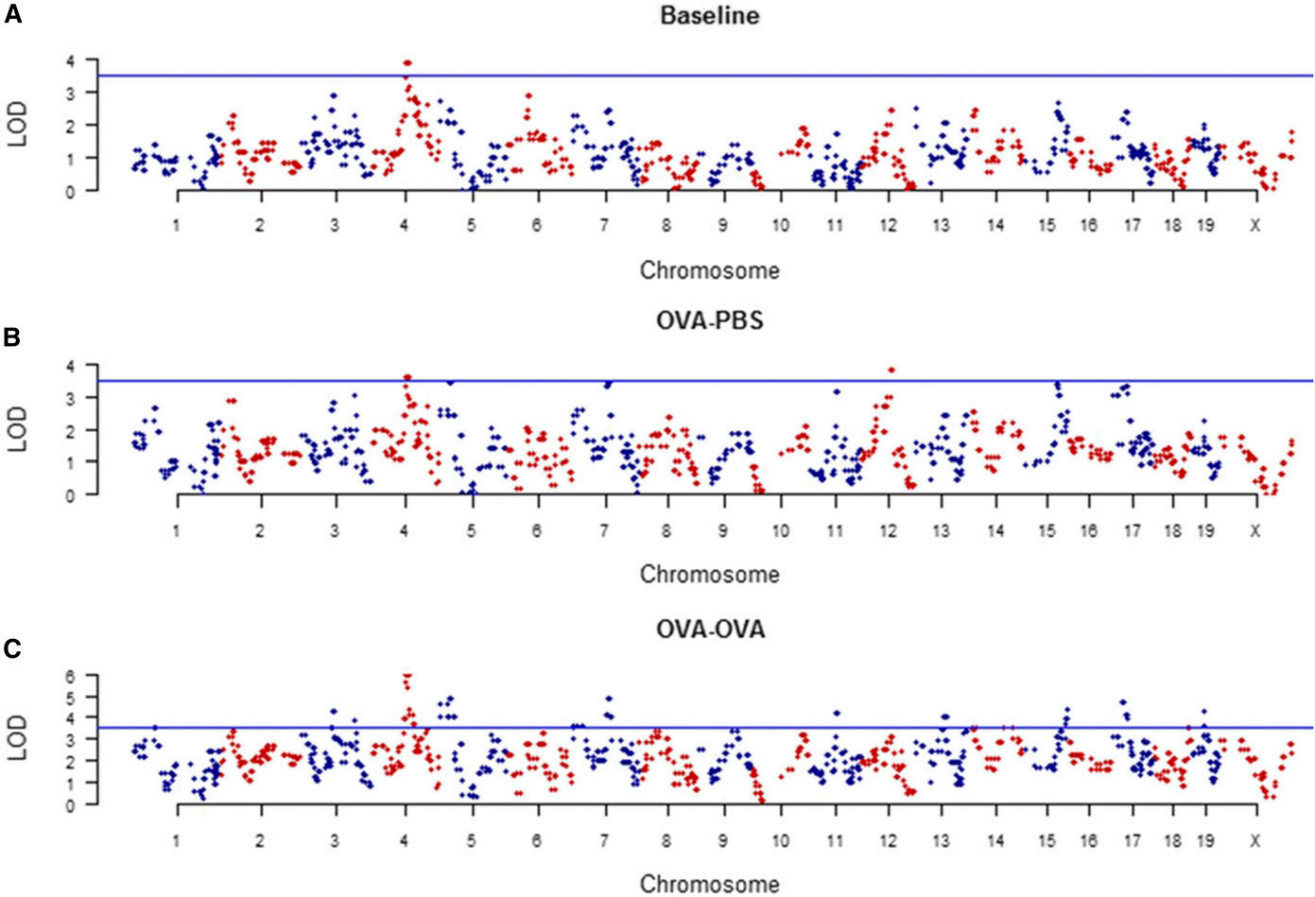
Identification of markers associated with the IgE phenotype at (A) baseline, (B) following ovalbumin sensitization and PBS challenge (OVA-PBS), and (C) ovalbumin sensitization and ovalbumin challenge (OVA-OVA) was done using a linear regression; significance thresholds were calculated after 10,000 permutations. Lines representing significance thresholds at α = 0.05 are presented for each graph (naïve = 3.498, OVA-PBS = 3.447, OVA-OVA = 3.598). Markers with *P*-values greater than the threshold were considered significant. Regions associated with the phenotypes are listed in Table 1.

To identify the chromosomal regions associated with plasma IgE levels, we performed a marker by marker linear regression analysis using the phenotype data, and genotype data of each strain at 1215 markers distributed across the genome (Boivin *et al.* 2012). Separate analyses were done for IgE levels at baseline, following ovalbumin sensitization and mock PBS challenge, and following ovalbumin sensitization and ovalbumin challenge. Markers that surpassed the significance threshold were considered associated with the phenotype (Figure 3). The regions delimited by the markers that were significant are summarized in Table 1. We observed an increase in the number of regions involved in the regulation of the IgE trait following allergen exposure (Figure 3). Interestingly, only one region on Chromosome 4 (81.46–86.17 Mbp) is significantly associated with the IgE phenotype at baseline, and is also associated with the phenotype following allergen sensitization with or without allergen challenge.

**Table 1:** Chromosomal regions significantly associated with circulating levels of plasma IgE in AcB/BcA panel of congenic mice

	Baseline		OVA-PBS		OVA-OVA	
Chr.	Region (Mbp)	Peak *P* Value	Region (Mbp)	Peak *P* Value	Region (Mbp)	Peak *P* Value
3					73.51–76.06	5.15 × 10^−5^
					121.46–126.41	1.25 × 10^−4^
4	81.46–86.17	1.17 × 10^−4^	81.46–86.17	2.53 × 10^−4^	75.06–101.43	1.08 × 10^−6^
5					3.06–34.77	1.24 × 10^−5^
7					3.07–29.93	2.46 × 10^−4^
					70.81–84.21	1.24 × 10^−5^
11					63.66–76.96	6.68 × 10^−5^
12			67.30–76.98	1.49 × 10^−4^		
13					67.76–73.80	8.67 × 10^−5^
15					96.69–103.45	3.96 × 10^−5^
17					26.61–41.02	2.0 × 10^−5^
19					26.75–28.74	5.34 × 10^−5^

Regions are based on reference “Genome Reference Consortium GRCm38,” UCSC version mm10. 5% significance thresholds are: Baseline: 3.17 × 10^−4^; OVA-PBS: 3.57 × 10^−4^; OVA-OVA: 2.52 × 10^–4^.

### Mouse Chromosome 4 and IgE

**Figure 4:**
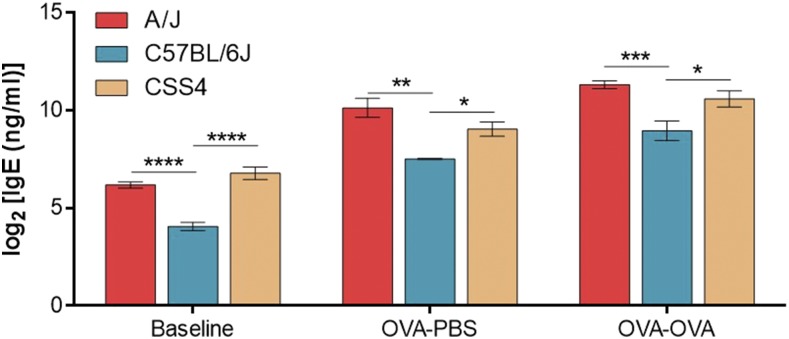
Plasma IgE concentration of Chromosome 4 substitution strain (CSS4) relative to parental strains A/J and C57BL/6J following different degrees of allergen exposure. Results are presented for unsensitized (Baseline), ovalbumin (OVA) sensitized and PBS challenged (OVA-PBS), and OVA sensitized and OVA challenged animals (OVA-OVA). Comparisons between strains for each phenotype was done by one-way ANOVA followed by Bonferroni correction. Data are presented as mean of log_2_ transformed values ± SEM *n* ≥ 5 per strain; *, **, and *** represent *P* < 0.05, *P* < 0.01, and *P* < 0.001, respectively.

Based on the marker association results, we hypothesized that mouse Chromosome 4 likely harbors one or more genes important in the control of plasma IgE levels. To test this hypothesis, we used CSS4 mice, which have a C57BL/6J background with Chromosome 4 from the A/J strain. Plasma IgE levels were measured at baseline, after ovalbumin sensitization, and after ovalbumin sensitization and aerosol challenge in the CSS4 mice. The results illustrated in Figure 4 demonstrate that substitution of A/J Chromosome 4 on a C57BL/6J background is sufficient to significantly increase the IgE phenotype within a range comparable to the A/J strain under all three tested conditions.

## Discussion

We were able to confirm the polygenic nature of the plasma IgE phenotype in our ovalbumin allergen-exposed mice through the identification of multiple genomic regions associated with the trait. On the other hand, the analysis of baseline IgE levels in the RCS mice identified only a single associated chromosomal region on Chromosome 4 (81.46–86.17 Mbp). This region was associated with IgE levels under all three conditions. From our experiments involving CSS4 mice, we understand that the Chromosome 4 locus alone does not recapture the entire complex trait. At baseline, A/J and CSS4 mice have similar differences from C57BL/6J mice in their IgE levels; however, in the allergen-exposed models (ovalbumin sensitization followed by PBS challenge, and ovalbumin sensitization followed by ovalbumin challenge) A/J mice have a larger difference from C57BL/6J mice than CSS4 mice. These findings support the supposition that the genetic factors influencing baseline IgE levels, the increase in levels due to antigen sensitization, and the further increase in levels due to antigen exposure, affect the phenotype in a stepwise manner at each level of allergen exposure in the process of developing an IgE-mediated allergic response.

In only two cases did we observe overlap between the IgE-associated regions identified in studies by other groups and our own. Ohno *et al.* 2013 detected a suggestive association between ovalbumin induced IgE and Chromosome 17 at 35 Mbp, which falls within the Chromosome 17 region we identified using our ovalbumin-sensitized and ovalbumin-challenged model (Ohno *et al.* 2013). Chromosome 4 (from 13.95 to 31.66 Mbp) was associated with house dust mite antigen induced IgE in the 151 incipient lines of the Collaborative Cross (Kelada *et al.* 2014). Differences in the mouse strains and allergens used in each study can account for the differences in linkage results. This lack of reproducibility in linkage results is also observed in human studies of complex traits, where different regions are identified in different populations.

The human region homologous to mouse Chromosome 4 from 81.46 to 86.17 Mbp is found on Chromosome 9 from 13.31 to 18.60 Mbp. While this region has not been previously associated with atopy, human Chromosome 9 has been associated with IgE levels in a prior study (Wjst *et al.* 1999). Our IgE-associated region contains 12 protein coding genes that could be explored as candidates for the phenotype, such *Bnc2* and *Psip1*, which are involved in immune system development, and are associated with other allergic immune disorders (Ochs *et al.* 2000; Moy *et al.* 2015). Mutation assays performed using the Mouse Genome Informatics database did not identify any mutations in the protein coding regions of these two genes. However, nonsynonymous mutations were identified in the coding sequences of *Cer1*, *Ttc39b*, *Ccdc171*, *Cntln*, *Adamtsl1*, *Haus6*, *Gm12551*, and *Dennd4c*, but these genes do not have any prior documented associations with allergy, IgE, or asthma (Eppig *et al.* 2012). Further studies exploring the functions of these genes and SNPs in the context of allergy need to be performed to validate their importance.

Plasma IgE concentration and airway responsiveness have often been shown to go hand-in-hand in allergic asthma (Burrows *et al.* 1989). However, no overlapping regions were identified in our current study on baseline IgE, and our previous study on baseline airway responsiveness (Camateros *et al.* 2010). Similar findings, *i.e.*, that AHR may be IgE independent since it can develop in B-cell and mast cell deficient mice, have also been observed by other groups (Korsgren *et al.* 1997; Takeda *et al.* 1997).

To our knowledge, no other studies have reported exploring the genetics of baseline IgE concentration, but instead have focused on IgE levels postallergen exposure (Badalova *et al.* 2002; Lipoldova *et al.* 2002; Menge *et al.* 2003; Ohno *et al.* 2013; Kelada *et al.* 2014). By studying the IgE phenotypes at baseline and following allergen exposure, we identified a highly significant region on Chromosome 4 common to both. The methods we used can be applied to study other asthma phenotypes, such as the recruitment of eosinophils to the lungs and the production of allergy-associated cytokines.

## Supplementary Material

Supplemental material is available online at www.g3journal.org/lookup/suppl/doi:10.1534/g3.117.042739/-/DC1.
